# Examining the incidence of catastrophic health expenditures and its determinants using multilevel logistic regression in Malawi

**DOI:** 10.1371/journal.pone.0248752

**Published:** 2021-03-31

**Authors:** Atupele N. Mulaga, Mphatso S. Kamndaya, Salule J. Masangwi

**Affiliations:** 1 Faculty of Applied Sciences, Department of Mathematics and Statistics, University of Malawi, Blantyre, Malawi; 2 Centre for Water, Sanitation, Health and Appropriate Technology Development (WASHTED), University of Malawi, Blantyre, Malawi; University of Botswana, BOTSWANA

## Abstract

**Background:**

Despite a free access to public health services policy in most sub-Saharan African countries, households still contribute to total health expenditures through out-of-pocket expenditures. This reliance on out-of-pocket expenditures places households at a risk of catastrophic health expenditures and impoverishment. This study examined the incidence of catastrophic health expenditures, impoverishing effects of out-of-pocket expenditures on households and factors associated with catastrophic expenditures in Malawi.

**Methods:**

We conducted a secondary analysis of the most recent nationally representative integrated household survey conducted by the National Statistical Office between April 2016 to 2017 in Malawi with a sample size of 12447 households. Catastrophic health expenditures were estimated based on household annual nonfood expenditures and total household annual expenditures. We estimated incidence of catastrophic health expenditures as the proportion of households whose out-of-pocket expenditures exceed 40% threshold level of non-food expenditures and 10% of total annual expenditures. Impoverishing effect of out-of-pocket health expenditures on households was estimated as the difference between poverty head count before and after accounting for household health payments. We used a multilevel binary logistic regression model to assess factors associated with catastrophic health expenditures.

**Results:**

A total of 167 households (1.37%) incurred catastrophic health expenditures. These households on average spend over 52% of household nonfood expenditures on health care. 1.6% of Malawians are impoverished due to out-of-pocket health expenditures. Visiting a religious health facility (AOR = 2.27,95% CI:1.24–4.15), hospitalization (AOR = 6.03,95% CI:4.08–8.90), larger household size (AOR = 1.20,95% CI:1.24–1.34), higher socioeconomic status (AOR = 2.94,95% CI:1.39–6.19), living in central region (AOR = 3.54,95% CI:1.79–6.97) and rural areas (AOR = 5.13,95% CI:2.14–12.29) increased the odds of incurring catastrophic expenditures.

**Conclusion:**

The risk of catastrophic health expenditures and impoverishment persists in Malawi. This calls for government to improve the challenges faced by the free public health services and design better prepayment mechanisms to protect more vulnerable groups of the population from the burden of out-of-pocket payments.

## Introduction

The goal of health care financing system is to protect households from the financial risk due illnesses. This goal is well articulated in the world health organization 2010 report as the Universal Health Coverage (UHC) goal [1]. The UHC goal ensures that all people have access to health services and do not face financial hardship due to out-of-pocket health payments [1]. One way in which health systems can protect households from financial burden due to out-of-pocket payments is through prepayment mechanisms [2]. In most low and middle income countries (LMICs) health prepayment mechanisms are not well developed and most households rely on out-of-pocket payments for health services [3]. Such reliance places financial burden on households which leads to catastrophic expenditure, impoverishment, prevents households from accessing health care and makes the attainment of the Universal Health Coverage difficult [1].

Catastrophic health expenditures (CHEs) occur when out-of-pocket health payment as a share of household’s income or capacity to pay exceeds a predetermined threshold level [4]. CHE pushes households into poverty and leaves members of the households in a vicious circle of poverty and ill health [5,6]. These effects are common in LMICs where many households rely on out-of-pocket for payment of health care services [4]. Multi-country studies showed that an estimated 118.7 and 531.1 million people from Africa and Asia respectively incurred CHEs and 14.9 and 79 million people respectively were impoverished due to health payments by 2010 [7,8]. African and Asian countries accounted for 3.3% of the population impoverished by out-of-pocket health payments [8]. These findings from multi-country studies advance the need to understand the extent of CHEs and its associated risk factors in LMICs to design strategies for financial protection at national level.

Malawian health system is mainly publicly financed through tax revenues and receives substantial funding from external donors [9]. A minimum package of health services is provided for free in all public health facilities through the essential health package (EHP). This acts as a priority setting tool and includes key public health priority areas and cost effective intervention to address the major causes of mortality and morbidity [10]. Total health expenditure in Malawi increased by 14.7% from MWK429.1 billion to MWK502.8 billion over the period 2015–2018 and the average total per capita expenditure over the period was US $39.8 slightly higher than US$ 39.2 reported over the 2012–2015 period. The total per capita expenditure US$39.8 reported is similar to the average total per capita expenditure of US$41 in other low income countries but 2 times lower than the recommended total per capita expenditures of US$ 80 per year by WHO to strengthen health systems and implement a minimum set of essential health interventions [11,12]. Further to that the percapita expenditures is 5 times lower than the Southern Africa Development cooperation average of USD$ 209 in 2018 [13]. Such low total per capita expenditures may hinder the country to provide a minimum essential health services and consequently hinder its progress toward universal health coverage. Over the 2015–2018 period external donors contributed 58.6% of total health expenditures while Government and private health expenditures represented 23.9% and 17.5% of total health expenditures respectively. Out of 17.5% of private health expenditures 12.6% were from household’s out-of-pocket expenditures [12]. This shows there was an increased in private health expenditures from 13.4% in 2012–2015 period to 17.5% of total expenditures mainly due to the rise in out-of-pocket expenditures from 8.6% of total health expenditures to 12.6% in the 2015–2018 period.

While access to health services in public facilities is free at point of use, households still contribute to total health expenditure through out-of-pocket payments in Malawi. Two main factors could explain this phenomenon. Firstly, the health system face many bottlenecks such as shortage of drugs, skilled medical personnel, poor quality of services and inaccessibility of facilities [14]. These bottlenecks force households to seek care in private health facilities with better quality services and skilled medical personnel where they incur higher out-of-pocket health payments. Shortage of drugs may also force households to purchase drugs at private pharmacies where they incur higher out-of-pocket payments. Secondly, in Malawi prepayment and risk pooling mechanisms for health financing are underdeveloped. Malawi has no social health insurance or health fund and for the private health insurance coverage is low and only accessible to those in the formal employment sector [15]. For instance, 1% of women and 2% of men aged 15–49 in the formal employment sector have health insurance coverage [15]. These low percentages suggest that higher costs of private health insurance leave many in the formal employment sector and those in the informal sector at a risk of catastrophic health expenditure, impoverishment and constrained when accessing health care.

According to previous studies, out-of-pocket health payments expose households to the risk of CHEs and impoverishment [5,16–21]. These studies also show that households in rural areas, in lower socioeconomic status, with chronically ill members, with children, with elderly members and larger households are at an increased risk of incurring CHEs. A study by Mchenga et al [22] showed that 0.73% to 9.73% of households faced CHEs in Malawi. The same study found that out-of-pocket expenditures increases the incidence of CHE and pushes households into poverty [22]. However, existing research in Malawi has paid limited attention to examining factors associated with CHEs. This paper compliments existing research by determining factors associated with CHEs using the most recent available fourth integrated household survey data (IHS4) in Malawi. We also examine the incidence, intensity of CHEs and the impoverishing effects of out-of-pocket expenditures on households. Our study provides evidence on the extent of CHEs and impoverishing effects of out-of-pocket health payments on households in a context of a country with a free public health services policy. It also provides evidence to policy makers on the characteristics of households that are vulnerable to CHEs. Such evidence is relevant in the designing of financial protection interventions in LMICs.

## Methods

### Data source

This study is a secondary analysis of data from a nationally representative integrated household survey (IHS4) conducted between April 2016 to April 2017 by the National Statistics office of Malawi [23]. The IHS4 used a stratified two stage sampling design. In the first stage,780 enumeration areas stratified by urban and rural strata were selected with probability proportional to size. The second stage used a random systematic sampling to select 16 primary households and 5 replacement households from the household listing in each sample enumeration area. A total of 12480 households were interviewed and data for 33 households were lost. Data for a total sample of 12,447 households covering 53,885 individuals were collected and this represented a 99.7% response rate. Our analysis used data for all the 12,447 households. The survey collected data on households’ economic activities, demographics, welfare and other household characteristics. Particularly, data on the health module collected information on health spending on illnesses and injury over one-month recall period, expenditures on hospitalizations at a health facility and at a traditional healer over twelve months’ recall period, chronic illnesses and diagnosis source of illnesses. The consumption expenditure module collected information on food expenditures and nonfood expenditures. The food consumption expenditures information collected over a one-week recall period included expenditures on items such as cereals, roots, tubers, nuts, pulses, vegetables, meat, fish, meat products, milk, milk products, fruits, sugar, fats, oils beverages and other miscellaneous items. For the nonfood consumption expenditure different recall periods were used for different items. Expenditures for items such public transport, charcoal, kerosene, cigarettes, newspapers and magazines were collected over one-week recall period. Expenditures for items including groceries, wages paid to servants, motor vehicle service, mortgage, repairs to household item were collected over one-month recall period and clothing over three-month period. Expenditures for items such as carpets, rugs, linen, sleeping mats, construction materials, council rates, funeral and marriage ceremony costs were collected over twelve months’ period. The aggregated data for all consumption expenditures were annualized and for consistency we report the findings for annual consumptions expenditures. All data including the food consumption expenditures data were collected using interviewer administered questionnaire.

### Ethical considerations

Ethical clearance for this secondary reanalysis was obtained from National Committee on Research in the Social Sciences and Humanities (NCRSH) reference No. P.10/19/434. The National Statistical Office of Malawi enumerators obtained verbal informed consent from the participants and this was recorded on the questionnaire and upon agreement to participate the enumerator proceeded with the interview.

### Data analysis

#### Measuring incidence and intensity of catastrophic health expenditures

To assess catastrophic health expenditures we used measures proposed by Wagstaff and Doorslaer [24]. Wagstaff and Doorslaer [24] proposed two indicators for assessing catastrophic health payments; these are catastrophic payment head count which measures the incidence and catastrophic payment overshoot which measures the intensity of catastrophic health payments.

Catastrophic health expenditure *E* is defined as [24,25]:

$$E=\left\{ \begin{array}{c} 1,\;if\frac{T}{x-f(x)}>Z\\ 0,\;otherwise \end{array}\right.$$ (1)

Where *x* is the total annual household’s consumption expenditure, *Z* is the threshold level, *T* is the total annual household’s out-of-pocket health payments and *f*(*x*) is the total annual household’s food expenditures.

Catastrophic payment head count denoted by *H*_*cata*_ was estimated as [25]:

$$H_{cata}=\frac{1}{N}\sum_{i=1}^{N}E_{i}=\mu_{E}$$ (2)

where N is the sample size.

The catastrophic payment overshoot is defined as $O_{i}=E_{i}\left[(\frac{T}{x-f(x)})-Z\right]$ [24,25].

Therefore, average catastrophic payment overshoot was estimated as [24,25]:

$$O_{cata}=\frac{1}{N}\sum_{i}^{N}O_{i}=\mu_{O}$$ (3)

where N is the sample size and the average overshoot in (3) measures the intensity of catastrophic health payments. The catastrophic mean positive gap(overshoot) denoted by MPG was estimated as:

$${MPG}_{cata}=\frac{O_{cata}}{H_{cata}}=\frac{\mu_{O}}{\mu_{E}}$$ (4)

In this study households incurred CHEs if out-of-pocket health expenditures as a share of household’s capacity to pay exceed 40%, where household’s capacity to pay was defined as annual household consumption expenditures remaining after food expenditures [4] and we also defined CHEs based on 10% threshold level of total consumption expenditures [24]. The choice of threshold levels is arbitrary however in the literature threshold levels of 40% of household capacity to pay and 10% of total consumption expenditures have been used [25]. In addition, CHEs defined based on 10% of total consumption expenditures is the official indicator for monitoring universal health coverage financial protection among the Sustainable Development Goals (SDGs indicator 3.8.2) [7,26]. For comparison of results we also reported findings on the incidence and intensity of CHEs for the threshold levels 20%, 25% and 30%. We defined out-of-pocket health expenditures as expenditures made at a point of use of health services [4]. We estimated out-of-pocket health expenditures as expenditures on consultation fees, diagnostic tests, medicines, outpatient and hospitalization fees.

#### Assessing impoverishing effects of out-of-pocket health expenditures on households

Impoverishment due to out-of-pocket payments occurs when non poor households become poor after paying for health services [24]. To assess the impoverishing effects of out-of-pocket health payments we examined the effects of health payments on two commonly used poverty measures; these are poverty headcount and poverty gap [24,25]. We estimated impoverishment impact due to health expenditures as the difference between post-payment poverty head count and pre-payment poverty headcount. Poverty head count gives the proportion of population with total consumption expenditures below the poverty line and poverty gap gives the extent by which the average total consumptions expenditures of the poor fall below the poverty line. We used the 2016 Malawi national poverty line of 137425 MWK [27] to examine the impoverishing effects of out-of-pocket payments.

Suppose we define $P_{i}^{pre}=\left\{ \begin{array}{l} 1,if\;x_{i}<PL\\ 0,\;otherwise \end{array}\right.$ where *PL* denotes the poverty line and *x*_*i*_ is the total annual household consumption expenditure per capita for household *i*; as the individual household *i* poverty before out-of-pocket health payments. Then the average pre-payment poverty headcount was estimated as [24,25]:

$$H_{poverty}^{pre}=\frac{1}{N}\sum_{i=1}^{N}P_{i}^{pre}=\mu_{p^{pre}}$$ (5)

where N is the sample size. We defined poverty gap before out-of-pocket health payments for each individual household *i* as $g_{i}^{pre}=P_{i}^{pre}(PL-x_{i}).$ Hence the average prepayment poverty gap was estimated as [24,25]:

$$G_{poverty}^{pre}=\frac{1}{N}\sum_{i=1}^{N}g_{i}^{pre}=\mu_{g^{pre}}$$ (6)

Where N is the sample size. The normalized poverty gap before health payments was estimated as:

$${NGap}^{pre}=\frac{G_{poverty}^{pre}}{PL}$$ (7)

We obtained similar measures for the post payment poverty head count and gap after subtracting total annual household’s out-of-pocket expenditure per capita from total annual household’s consumption expenditure per capita and replacing the superscripts in Eqs 5,6 and 7 with post payment.

The difference between the corresponding post and pre poverty measures gives the impoverishing effects of out-of-pocket health payments on households. For example, we estimated the impoverishing effects of out-of-pocket payments on poverty head count and gap using the differences:

$${PI}_{headcount}=H_{poverty}^{post}-H_{poverty}^{pre}\;\mathrm{and}\;{PI}_{gap}=G_{poverty}^{post}-G_{poverty}^{pre}$$

#### Assessing factors associated with catastrophic health payments

A multilevel binary logistic regression was used to assess the factors associated with catastrophic health expenditures. This regression was used to account for the nested structure of the survey data where households are nested in districts and to ensure correct estimation of standard errors and statistical inference of the model parameters. This binary regression was also used to account for our main outcome variable which takes the value of 1 if a household incurred catastrophic health expenditure and zero otherwise. We estimated two models; model 1 was estimated with CHEs defined based on 40% of household nonfood consumption expenditures and model 2 with CHEs based on 10% of household total consumption expenditures.

*Multilevel binary logistic regression model*. Let *Y*_*ij*_ be the outcome of catastrophic health expenditures for the *i*^*th*^ household in *j*^*th*^ district, *π*_*ij*_ be the probability of incurring catastrophic health expenditures and *x*_*ij*_ be some household level covariates. We assume *Y*_*ij*_ follows a binomial distribution, i.e. *Y*_*ij*_~*Bin*(1,*π*_*ij*_). Then, the probability of incurring catastrophic health expenditures *π*_*ij*_ is modelled using a logit link function and the random intercept model is specified as:

$$logit(\pi_{ij})=\beta_{0}+\beta x_{ij}+u_{j}$$ (8)

Where *β* is a vector of fixed effects regression coefficients of the corresponding household level covariates *x*_*ij*_ and *u*_*j*_ is the district level random effects term which captures the unobserved district level effects. The district level random effects term is assumed to be normally distributed with mean of zero i.e. $u_{j}∼N(0,\sigma_{u}^{2})$.

We included as covariates those factors identified in previous literature as determinants of catastrophic health expenditures [5,16–21]. These included household head characteristics such as age in years, sex, education and other household characteristics such as household size, socioeconomic status, presence of at least one chronically ill member in the household, presence of at least one elderly member, presence of at least one child, presence of at least one hospitalized member over the past 12 months, location of household(rural/urban), region in which the household is located, distance to the nearest health facility with a medical doctor and type of health facility with medical doctor. The measure of socioeconomic status was constructed based on total household consumption expenditure per capita. Total consumption expenditure per capita was categorized into five consumption expenditure quintiles from the poorest to the richest quintile. Data analysis was done using Stata 15. All analyses were adjusted for sampling design using survey sample weights and the survey set command in Stata 15. All results were interpreted at 5% significance level.

## Results

### Socio-economic and demographic characteristics of the sampled households

Table 1, shows the socio-economic and demographic characteristics of the sampled households. About 27% of the household heads were 26 to 35 years old and a larger majority of the households (71.12%) were male headed. About 63% of the household heads had no formal education, 83.32% were unemployed and only 2.34% received social safety nets from government. A larger proportion (80.95%) of the households was rural. More than half of the households (53.51%) had children under the age of five years old and about 20% of the households had members older than 60 years old. A smaller proportion (13.16%) of the households had at least one member hospitalized and 22.33% had at least one member with chronic illnesses such as diabetes, tuberculosis, HIV/AIDS and arthritis. The average household size was four. A larger proportion (87.23%) reported having a nearest medical doctor at a government health facility. The average distance to nearest health facility with a medical doctor was 13 kilometers.

**Table 1:** Socio-economic and demographic characteristics of the sampled households (n = 12447).

Variable	Mean(SD) or %
Age of household head	
Less than 26 years	12.30
26–35 years	26.66
36–45 years	23.79
46–55 years	15.21
Over 56 years	22.04
Male headed household	71.12
Education level of household head	
None	63.16
Primary	12.60
Secondary	19.80
Tertiary	4.44
Household head Employed	16.68
Household received social safety nets	2.34
Household size	4.29(2.00)
Presence of at least one child under 5 years	53.52
Presence of at least one elderly member greater than 60 years	19.75
Presence of at least one chronically ill member	22.33
Presence of at least one hospitalized member	13.16
Rural household	80.95
Distance to the nearest health facility with medical doctor (KM)	13.33(16.85)
Type of health facility from which medical doctor is based	
Government	87.23
Religious	10.68
Private	2.08
Region	
Northern	9.15
Central	44.32
Southern	46.53

More households (32.12%) from the fourth income group and 30.63% of the households from rural reported illnesses in the past two weeks preceding the survey as shown in Table 2.

**Table 2:** Percentage of households reporting illnesses over 2 weeks’ recall period by SES, location of household and region(n = 12440).

Variable	No of households reporting illnesses	Total no. of households	% of households reporting illnesses (95% CI)
Socio-economic status			
Quintile 1(Poorest)	655	2504	26.16 (24.23–28.78)
Quintile 2	724	2473	29.28 (28.23–32.89)
Quintile 3	741	2478	29.90 (28.73–33.28)
Quintile 4	784	2441	32.12 (29.67–34.49)
Quintile 5(Richest)	758	2544	29.44 (28.09–32.79)
Location of household			
Urban	546	2268	24.07 (21.61–27.35)
Rural	3116	10172	30.63(29.99–32.86)
Region			
Northern	582	2488	23.39(20.77–25.08)
Central	1372	4218	32.53(30.66–34.89)
Southern	1708	5734	29.79(27.14–30.81)

Table 3, presents household annual out-of-pocket health payments on medicine, out-patient care and hospitalizations by socio-economic status and location. Overall, the average total annual out-of-pocket health payment for all households was MWK15648.78. The mean total annual out-of-pocket health payment for drugs was MWK 5488.84, MWK 8412.35 for out-patient services and MWK 1747.58 for hospitalizations. A larger amount of a total annual out-of-pocket health payment was spent on out-patient services, this expenditure on out-patient services represented over half (53.75%) of the total out-of-pocket health payments. Households in the richest income quintile spent more on drugs(7745.14MWK), out-patient services(18528.38MWK) and hospitalizations(3427.57MWK) compared to poorest households. Overall, the mean out-of-pocket health spending for richest households was significantly higher (29701.1 MWK) compared to MWK 6480.44 for poorest households. Total annual out-of-pocket health payment was higher (MWK 23291.92) among households in urban compared to rural (MWK13850) areas and was higher in households in central region compared to northern and southern regions.

**Table 3:** Households out-of-pocket health payments by SES, location of household and region.

Variable	Mean annual out-of-pocket health payments in Malawi Kwacha (MKW)			
	Drugs	Out-patients	Hospitalizations	Total health payments
Socio-economic status				
Quintile 1(Poorest)	3374.11 (2919.87–3828.34)	2185.45 (1567.33–2803.56)	920.89 (724.09–1117.68)	6480.44 (5506.942–7453.942)
Quintile 2	4548.75 (3984.87–5112.64)	4545.97 (3473.23–5618.71)	1393.13 (1110.89–1675.36)	10487.85 (9010.388–11965.31)
Quintile 3	5085.307 (4356.13–5814.48)	6932.79 (5359.91–8505.69)	1303.59 (1007.08–1600.11)	13321.7 (11348.03–15295.37)
Quintile 4	6692.28 (5808.35–7576.19)	9877.10 (7839.55–11914.66)	1693.99 (1343.35–2044.63)	18263.37 (15574.03–20952.72)
Quintile 5(Richest)	7745.14 (6673.76–8816.53)	18528.38 (15009.45–22047.32)	3427.57 (2348.76–4506.39)	29701.10 (24847.66–34554.53)
Location of household				
Urban	6536.27 (5277.23–7795.32)	13589.04 (10012.57–17165.51)	3166.61 (2055.82–4277.39)	23291.92 (18033.83–28550.01)
Rural	5242.33 (4718.81–5765.85)	7194.05 (6010.52–8377.59)	1413.621 (1240.98–1586.27)	13850 (12213.51–15486.5)
Region				
Northern	5570.08 (4631.88–6508.28)	7935.04 (5029.32–10840.75)	1652.61 (1299.11–2006.10)	15157.72 (11489.09–18826.36)
Central	6657.99 (5753.26–7562.72)	11844.2 (9660.37–14028.03)	1748.88 (1457.74–2040.02)	20251.07 (17291.58–23210.56)
Southern	4359.24 (3836.65–4881.83)	5237.4 (4037.11–6437.69)	1765.03 (1288.13–2241.92)	11361.66 (9404.162–13319.17)
All households	5488.84 (5002.78–5975.31)	8412.35 (7239.78–9584.94)	1747.58 (1491.02–2004.14)	15648.78 (13989.75–17307.81)

95% CI in parenthesis.

Table 4 gives results on out-of-pocket expenditures as a share of total household expenditures per capita by consumption expenditure quintiles and the Kakwani indices to measure progressivity of out-of-pocket payments. Overall the share of total out-of-pocket health expenditures as percentage of total household expenditure decrease with increase in total expenditures, indicating that out-of-pocket health expenditures are regressive. The share of expenditures on drugs and hospitalizations as a percentage of total household expenditures decrease with increase in total household expenditure indicating that expenditures on drugs and hospitalizations are regressive. Results of the Kakwani index provide similar conclusions. All the Kakwani indices are negative which implies that out-of-pocket health expenditures are regressive as poor households contributes a larger share of their income in paying for health services than rich households.

**Table 4:** Out-of-pocket payments as a share of total household expenditures per capita by expenditure quintiles (%).

Expenditure quintile	Drugs	Outpatients	Hospitalizations	Total health expenditures
Quintile 1(Poorest)	4.70	3.01	1.33	9.04
Quintile 2	4.13	4.04	1.26	9.42
Quintile 3	3.42	4.16	0.87	8.90
Quintile 4	3.23	4.67	0.83	8.73
Quintile 5(Richest)	2.09	4.39	0.78	7.26
Kakwani index	-0.29***	-0.06	-0.19*	-0.16*

Note

*** significant at 1%

* * significant at 5%

* significant at 10%. Kakwani index measures the progressivity in health finance and lies between -2(most regressive financing) and +1(most progressive financing) [25].

Table 5, reports results of the incidence and intensity of catastrophic health expenditures as measured by catastrophic headcount and overshoot respectively. Incidence(headcount) and intensity(overshoot) of catastrophic health expenditures decrease with increase in the threshold level. Overall,1.37% and 4.14% of the households incurred catastrophic health payments at a threshold level of 40% of nonfood expenditures and 10% of total consumption expenditures respectively. The mean positive overshoot (MPO) was 12.71% at 40% of nonfood expenditures. Households that incurred catastrophic health payments at 40% of nonfood expenditures, on average spent over half (52.71%) of total nonfood expenditure on health care.

**Table 5:** Incidence and intensity of catastrophic health expenditures.

Catastrophic health expenditures measures			Threshold levels z (%)		
Out-of-pocket health payments as share of non-food expenditures	10%	20%	25%	30%	40%
Headcount (H)	14.08	5.83	3.99	2.84	1.34
Standard error for H	0.62	0.40	0.32	0.27	0.18
Overshoot (O)	1.68	0.78	0.54	0.37	0.17
Standard error for O	0.12	0.08	0.06	0.05	0.03
Mean positive Overshoot (MPO)	11.96	13.42	13.58	13.16	12.71
Standard error	0.48	0.67	0.78	0.87	0.88
		Threshold levels z (%)			
Out-of-pocket health payments as share of total expenditures	10%	20%	25%	30%	40%
Headcount (H)	4.14	1.31	0.84	0.48	0.11
Standard error for H	0.31	0.17	0.13	0.09	0.04
Overshoot (O)	0.35	0.12	0.07	0.04	0.01
Standard error for O	0.04	0.02	0.01	0.01	0.01
Mean positive Overshoot (MPO)	8.54	8.99	8.02	7.29	8.23
Standard error	0.51	0.76	0.91	1.19	1.96

The incidence of catastrophic health expenditures varied by socio-economic status, location of the household, type of health facility and type of health service utilized as shown in Table 6. Catastrophic health expenditures were high for households in rural areas (1.57%) compared to urban (0.38%), households in middle income groups and for households in the central region (2.09%) compared to southern and northern regions. CHEs were also higher among households utilizing religious facilities (2.32%) and outpatient services (11.34%).

**Table 6:** Incidence and intensity of catastrophic health expenditures: By SES, location of household (urban/rural), region, type of facility and type of health service utilized.

Variable	Incidence of CHE: threshold level z = 40 nonfood expenditures	Intensity of CHE: threshold level z = 40 nonfood expenditures	Incidence of CHE: threshold level z = 10% total health expenditures	Intensity of CHE threshold level z = 10% total health expenditures
Socio-economic status				
Quintile 1(Poorest)	0.74(0.36–1.52)	0.09 (0.03–0.14)	3.59(2.70–4.76)	0.25(0.15–0.35)
Quintile 2	1.54(1.03–2.29)	0.15 (0.07–0.22)	4.54(3.59–5.73)	0.34(0.23–0.45)
Quintile 3	1.65(1.09–2.48)	0.24 (0.12–0.37)	3.70(2.84–4.81)	0.39(0.24–0.54)
Quintile 4	1.53(0.98–2.39)	0.19 (0.09–0.28)	4.09(3.15–5.29)	0.36(0.23–0.48)
Quintile 5(Richest)	1.23(0.78–1.92)	0.19 (0.08–0.03)	4.77(3.66–6.18)	0.43(0.27–0.58)
Location of household				
Urban	0.38(0.16–0.86)	0.03 (0.01–0.05)	2.57(1.64–4.01)	0.19(0.09–0.29)
Rural	1.57(1.18–2.06)	0.20 (0.14–0.27)	4.51(3.86–5.26)	0.39(0.30–0.48)
Region				
Northern	0.73(0.42–1.27)	0.08 (0.03–0.14)	3.09(2.17–4.39)	0.22(0.12–0.31)
Central	2.09(1.47–2.96)	0.27 (0.16–0.38)	5.67(4.67–6.89)	0.52(0.38–0.67)
Southern	0.74(0.49–1.11)	0.09 (0.05–0.14)	2.88(2.25–3.68)	0.22(0.15–0.29)
Type of facility				
Government	1.28(0.94–1.75)	0.15(0.09–0.21)	3.96(3.35–4.67)	0.34(0.25–0.42)
Religious	2.32(1.42–3.78)	0.35(0.15–0.55)	6.13(4.38–8.51)	0.54(0.29–0.78)
Private	0.09(0.12–0.72)	0.03(0.02–0.09)	2.71(0.71–9.74)	0.19(0.05–0.45)
Type of service utilization				
Out patient	11.34(8.48–15.02)	1.57(1.01–2.13)	33.16(28.54–38.13)	3.31(2.58–4.04)
Inpatient	7.03(4.63–10.54)	0.88(0.41–1.36)	16.85(12.89–21.71)	1.79(1.06–2.52)

Note: 95% CI in parenthesis.

### Impoverishing effects of out-of-pocket health expenditures on households

Table 7, presents results on the impoverishing effects of out-of-pocket health expenditures on households. The poverty head count based on nonfood consumption expenditure was 51.53% and subtracting health expenditures from the nonfood expenditure the poverty headcount increased to 53.13%. This implies that over half (51.53%) of the population is considered living below the national poverty line of 137425MWK based on household nonfood consumption expenditures however when out-of-pocket health expenditures are accounted for, about 53.13% of the population is considered poor. This represented a 3.10% relative increase in the incidence of poverty. The poverty gap increased from MWK 23101.75 to MWK 24167.55 after subtracting health expenditures. The mean positive gap increased from 32.62% to 33.10% representing a 1.47% relative increase in the intensity of poverty after accounting for out-of-pocket health expenditures. The increase in the mean positive gap implies that the rise in the poverty gap is as a result of households that were already poor being pushed deeper into poverty due out-of-pocket health expenditures.

**Table 7:** Poverty effects of out-of-pocket health expenditures in Malawi, using the national poverty line (MWK137425).

	Gross of health payments	Net of health payments	Difference	
	(1)	(2)	Absolute (3) = (2)-(1)	Relative [(3)/(1)]*100
Poverty head count (%)	51.53	53.13	1.60	3.10
Poverty gap (MWK)	23101.75	24167.55	1065.80	4.61
Normalized poverty gap (%)	16.81	17.59	0.78	4.64
Normalized mean positive gap (%)	32.62	33.10	0.48	1.47

### Factors associated with catastrophic health expenditures

Table 8, presents results of the multilevel logistic regression models to assess the factors associated with the incidence of catastrophic health expenditures. The estimated district level random effects were significant indicating variations in CHEs between districts. The district level random effects explained 19% of the variation in CHEs. Several factors were associated with the risk of CHEs. We present results with CHEs defined based on 40% of nonfood expenditures. Households with more members had an increased odds of incurring catastrophic health expenditures (OR = 1.20, CI = 1.08–1.34). Having at least one household member hospitalized increased the odds of CHEs (OR = 6.03, CI = 4.08–8.90). Households headed by young household heads had a reduced odds of incurring CHEs. For example, households with households’ heads who were in the 46 to 55 age group had a 43% less odds of incurring CHEs than households headed by household heads who were over 56 years old (OR = 0.43, CI = 0.19–0.99). Higher socioeconomic status increased the odds of incurring catastrophic health expenditures. For example, households in the richest income quintile had 2.94 times greater odds of incurring catastrophic health expenditures (OR = 2.94, CI = 1.39–6.19) compared to households in the poorest income quintile. Location of the household increased the odds of incurring catastrophic health expenditures. For instance, Households in rural areas had 5.13 times more odds of incurring catastrophic expenditures (OR = 5.13, CI = 2.14–12.29) compared to urban households and households in central region had 3.54 times more odds of incurring catastrophic health expenditures (OR = 3.54, CI = 1.79–6.97). Having the nearest medical doctor based at a religious health facility increased the odds of incurring catastrophic health expenditures compared to having nearest medical doctor based at a government health facility (OR = 2.27, CI = 1.24–4.15).

**Table 8:** Multilevel logistic regression model for probability of incurring catastrophic health expenditures.

Independent variables	Model 1(CHE using 40% of non-food expenditures)	Model 2 (CHE using 10%of total health expenditures)
	Odds Ratio (95% CI)	Odds Ratio (95% CI)
Age of household head(ref = Over 56 years)		
Less than 26 years	0.44(0.17–1.15)	0.68(0.41–1.13)
26–35 years	0.59(0.27–1.32)	0.90(0.58–1.39)
36–45 years	0.52(0.23–1.10)	0.57(0.37–0.89)*
46–55 years	0.43(0.19–0.99)*	0.62(0.39–0.99)*
Sex of household head (ref = Male)	1.16(0.75–1.77)	1.04(0.82–1.32)
Household size	1.20(1.08–1.34)*	1.09(1.02–1.15)*
Socio-economic status (ref = Quintile 1(Poorest))		
Quintile 2	2.08(1.09–3.95)*	1.17(0.85–1.61)
Quintile 3	2.61(1.37–4.97)*	1.07(0.77–1.49)
Quintile 4	2.69(1.37–5.29)*	1.32(0.94–1.85)
Quintile 5(Richest)	2.94(1.39–6.19)*	1.89(1.33–2.70)*
Presence of at least one child (ref = No)	1.16(0.72–1.87	1.22(0.94–1.85)
Presence of at least one elderly member (ref = No)	0.73(0.34–1.53)	1.01(0.67–1.53)
Presence of at least one chronically ill member (ref = No)	1.40(0.94–2.11)	1.37(1.09–1.70)*
Presence of a hospitalized member(ref = No)	6.03(4.08–8.90)*	4.82(3.91–5.95)*
Household location (ref = Urban)	5.13(2.14–12.29)*	2.09(1.30–2.32)*
Distance to the nearest health facility with medical doctor	0.99(0.97–1.00)	1.00(0.99–1.01)
Type of facility utilized doctor based at (ref = government facility)		
Religious health facility	2.27(1.24–4.15)*	1.74(1.30–2.32)*
Private health facility	0.51(0.05–5.34)	1.65(0.83–3.29)
Region (ref = Northern)		
Central	3.54(1.79–6.97)*	2.59(1.46–4.59)*
Southern	1.09(0.54–2.22)	1.10(0.63–1.91)
District level random effects		
$\sigma_{u}^{2}$	0.61(0.24–1.54)*	0.24(0.11–0.49)*

Note

* indicates significant at 95% confidence interval.

## Discussion

This study assessed the incidence of catastrophic health expenditures, its determinants and the impoverishing effects of out-of-pocket health expenditures using the most recent Malawi fourth integrated household survey. Our study shows that out-of-pocket health expenditures are regressive as poorer households bear more financial burden relative to their income than richer households in Malawi. This study also shows that CHEs and impoverishment appear to have increased by 37% and 60% respectively since the last integrated household survey in 2010/11 [22]. This increase suggests that more people continue to be pushed into poverty and experience disruptions in living standards due to out-of-pocket payments despite government efforts for a free public health services policy to increase financial protection. There is need for the Malawi government to protect households from the financial burden through other equitable means of financing health such as mandatory health insurance. The level of CHEs at 40% of non-food expenditures in Malawi is similar to what was reported in Lesotho [28], both of which are within the Sothern Africa Development Community, but lower than what was observed in most Sub Saharan African countries [16,17,29–31].

The low levels of overall incidence of catastrophic health expenditures may not necessarily mean high levels of financial protection considering the fact that the Malawian health care financing system is not as well developed as other sub-Saharan African countries such as Kenya, Rwanda, Tanzania and Ghana [32] with higher incidence of catastrophic health expenditures. The low levels of CHEs may reflect poor households in ability to afford care due to high costs; this forces such households to forgo treatment to avoid the consequences of out-of-pocket health payments and are not counted as incurring CHEs [4,33,34]. Estimates from the data used in this study show that 4.98% of those who reported illnesses did not seek care due to financial reasons. Moreover, our findings on CHEs by income show that households in poorest income quintile incur lower incidence of CHEs and are at a decreased risk of facing CHEs compared to middle and richer income households. Though this finding is contrary from findings by previous studies [16,17,35–38] a possible explanation could be the challenges faced by free public health services in Malawi such as poor quality of services, shortages of drugs and poor attitude of medical personnel which forces households in the middle and richer income groups to seek better health care in private facilities and incur greater out-of-pocket health expenditures. On the other hand, inability of poor households to afford better health care from private facilities due to high costs may force them to forgo seeking health care. Government plans to establish a mandatory national health insurance scheme and a health fund financed through tax revenues [9] should be pursued. This coupled with improved services in public health facilities will ensure that all households have access to care and do not have to forgo care due to financial hardships.

We found that rural households incur high incidence of CHEs and are at an increased risk of CHEs as reported by other authors [16,17,28,32,39]. Rural households in Malawi are burdened with out-of-pocket expenditures due to poverty and high transportation costs in seeking care as health facilities in rural areas are far apart. As such even the little out-of-pocket expenses incurred on health care are catastrophic. Though our study did not assess the impact of other direct costs related to seeking health care such as transportation costs; estimates of the mean distance to the nearest health facility with a medical doctor using the data show that on average rural households travel about 17 KMs to seek health care compared to 4 KMs by urban households. In addition, most health facilities in rural areas are privately owned by religious institutions that charge user fees at point of use; higher health care costs puts households at a risk of CHEs and creates a barrier in financial protection among rural households [40]. This implies that policies that aim at increasing financial protection among rural households should also aim at reducing poverty and improving accessibility of health services in rural areas.

Our finding that hospitalizations increased the incidence of catastrophic health expenditures is consistent with findings from other studies [5,20,21,41,42]. Households with hospitalized members may sell assets, use savings and hire external labor as coping mechanisms. A study on coping with out-of-pocket payments in 15 African countries found that households with inpatient expenditures are more likely to sell assets and borrow as a means of coping with bills due to hospitalizations [43]. These coping strategies puts pressure on the household limited resources and leads to risk of CHEs.

The result that having the nearest medical doctor based at a religious health facility increased the odds of incurring CHEs than government facility is intuitive in the Malawian context. Religious health facilities charge user fees at point of use this implies households that access care at religious facilities are burdened with higher out-of-pocket payments. This finding corroborates with findings from Kenya [44]. For example, visiting a mission hospital increased the odds of incurring catastrophic health payments in Kenya [44]. The government of Malawi signed contracts called Service Level Agreements (SLAs) with mission health facilities in 2005 to ensure that households have access to services at these mission facilities without facing financial hardship [45]. Despite other studies showing that service level agreements improved utilization of health services [45] our finding may suggest that it has not achieved one of its intended purpose of protecting households from the financial burden of health expenditures. This is because many of the mission facilities and needed services are not part of the agreements and the poor who access services at these facilities still incur higher out-of-pocket payments [46]. There is need for government to expand these Service Level Agreements to include more facilities and services needed by households. This innovative financing mechanism has the potential to ensure many households have access to the needed health care without facing financial hardship [47].

The study has some limitations. Firstly, the study used self-reported data on consumption expenditures and illnesses which is prone to recall bias which can lead to underreporting as also observed by other authors. This limitation would underestimate the incidence of catastrophic health expenditures and impoverishing effects of out-of-pocket expenditures on households. Secondly, use of cross-sectional data prevents causal interpretation of the relationship between catastrophic health payments and its associated factors. Thirdly, data on total health expenditures were annualized this could lead to overestimating of total health spending as we assume the same rate of monthly health expenditures over time. Despite these limitations, our study makes use of a multilevel logistic regression model to assess factors associated with incidence of CHEs which highlighted variations by districts. In addition, the study assessed the incidence of CHEs, the impoverishing impact of out-of-pocket health payments on households using the most recent available data which is important for monitoring financial protection.

## Conclusion

Our results are important for monitoring the incidence of catastrophic expenditures and impoverishing effects of out-of-pocket health expenditures in Malawi consequently progress towards achieving Universal Health Coverage. Despite a free public health care policy, our findings suggest that the incidence of catastrophic health expenditures and impoverishment effect of out-of-pocket health expenditure has increased compared to a previous study using similar data. Our finding that rural households face high incidence of catastrophic payments reflects challenges faced by free public health facilities in providing much needed care to households considering that majority of rural population access free public health services. This finding calls for government to improve the challenges faced by free public health services to protect majority rural poor from the financial risk of out-of-pocket payments. This study also shows that access to medical doctors from religious health facilities, living in rural areas and hospitalizations increased the odds of incurring catastrophic payments. There is a need for government to establish more equitable health financing mechanisms such as a mandatory national health insurance scheme or a health fund and expand the innovative financing mechanism of Service Level Agreements with mission health facilities. This will ensure that the identified vulnerable groups of the population are protected from financial hardship due to out-of-pocket payments.
