# Correction: Examining the incidence of catastrophic health expenditures and its determinants using multilevel logistic regression in Malawi

**DOI:** 10.1371/journal.pone.0259090

**Published:** 2021-10-20

**Authors:** Atupele N. Mulaga, Mphatso S. Kamndaya, Salule J. Masangwi

There are errors in the Funding statement. The correct Funding statement is as follows: ANM was supported by the Consortium for Advanced Research Training in Africa (CARTA). CARTA is jointly led by the African Population and Health Research Center and the University of the Witwatersrand and funded by the Carnegie Corporation of New York (Grant No—G-19-57145), Sida (Grant No:54100113), Uppsala Monitoring Centre and the DELTAS Africa Initiative (Grant No: 107768/Z/15/Z). The DELTAS Africa Initiative is an independent funding scheme of the African Academy of Sciences (AAS)’s Alliance for Accelerating Excellence in Science in Africa (AESA) and supported by the New Partnership for Africa’s Development Planning and Coordinating Agency (NEPAD Agency) with funding from the Wellcome Trust (UK) and the UK government. The statements made and views expressed are solely the responsibility of the Fellow. The funders had no role in study design, data collection and analysis, decision to publish, or preparation of the manuscript.
